# The Effect of Statins on Mortality of Patients With Chronic Kidney Disease Based on Data of the Observational Medical Outcomes Partnership Common Data Model (OMOP-CDM) and Korea National Health Insurance Claims Database

**DOI:** 10.3389/fneph.2021.821585

**Published:** 2022-02-02

**Authors:** Ji Eun Kim, Yun Jin Choi, Se Won Oh, Myung Gyu Kim, Sang Kyung Jo, Won Yong Cho, Shin Young Ahn, Young Joo Kwon, Gang-Jee Ko

**Affiliations:** ^1^ Department of Internal Medicine, Korea University Guro Hospital, Seoul, South Korea; ^2^ Department of Internal Medicine, Korea University College of Medicine, Seoul, South Korea; ^3^ Biomedical Research Institute, Korea University Guro Hospital, Seoul, South Korea; ^4^ Department of Internal Medicine, Korea University Anam Hospital, Seoul, South Korea

**Keywords:** statins, chronic kidney disease, all-cause mortality, cardiovascular mortality, common data model, population-based study

## Abstract

The role of statins in chronic kidney disease (CKD) has been extensively evaluated, but it remains controversial in specific population such as dialysis-dependent CKD. This study examined the effect of statins on mortality in CKD patients using two large databases. In data from the Observational Medical Outcomes Partnership Common Data Model (OMOP-CDM) from two hospitals, CKD was defined as an estimated glomerular filtration rate < 60 mL/min/m^2^; we compared survival between patients with or without statin treatment. As a sensitivity analysis, the results were validated with the Korea National Health Insurance (KNHI) claims database. In the analysis of CDM datasets, statin users showed significantly lower risks of all-cause and cardiovascular mortality in both hospitals, compared to non-users. Similar results were observed in CKD patients from the KNHI claims database. Lower mortality in the statin group was consistently evident in all subgroup analyses, including patients on dialysis and low-risk young patients. In conclusion, we found that statins were associated with lower mortality in CKD patients, regardless of dialysis status or other risk factors.

## Introduction

Chronic kidney disease (CKD) is an emerging global health issue, which has increased in prevalence along with metabolic diseases such as diabetes and hypertension (1). After a decrease in renal function, CKD is accompanied by the accumulation of uremic toxins, biochemical alterations, and chronic inflammation; these metabolic alterations lead to increases in all-cause and cardiovascular mortality (2). The incidence of cardiovascular disease (CVD) is 1.4–3.4-fold greater in CKD patients than in the general population (3); the prevalences of dyslipidemia and atherosclerosis, which are major risk factors for CVD, are also greater in CKD patients (4). The management of these risk factors for CVD in CKD patients is essential; lifestyle changes and the use of statins (i.e., first-line treatment for hyperlipidemia in the general population) are recommended (5). Statins block the conversion of β-hydroxy β-methylglutaryl-CoA to mevalonate during cholesterol synthesis in hepatocytes; they also increase the number of low-density lipoprotein (LDL) receptors on the cell surface to reduce serum LDL cholesterol, with an excellent lipid-lowering effect (6). Furthermore, the anti-inflammatory action of statins has recently been studied (7, 8). Although various studies, including randomized controlled trials, have been published regarding the use of statins in CKD patients, the results in specific population of CKD such as dialysis dependent CKD or young-aged low risk CKD have been inconsistent. In this study, we examined the protective effect of statins with data from two hospitals using a common data model, then confirmed the findings using a national claims database; we aimed to establish in-depth and concrete evidence for the cardiovascular effects of statins in CKD patients.

## Materials and Methods

### Study Design

This was a multicenter, retrospective, and observational cohort study, based on data from the Observational Medical Outcomes Partnership Common Data Model (OMOP-CDM) database in two tertiary hospitals (Korea University Anam and Guro Hospitals [KUAH and KUGH, respectively]). The Observational Health Data Sciences and Informatics collaboration provides the OMOP-CDM schema, which is a system for using the electronic health records of each hospital as standardized structures of the OMOP-CDM database (https://github.com/OHDSI/CommonDataModel/) (9). Using the electronic health records databases of both hospitals, data were extracted, transformed, and loaded into the OMOP-CDM, with data mapped to a unique concept identifier (ID) using the internationally accepted International Classification of Diseases-10 (ICD-10) code system for diagnosis (10). For each hospital, data from February 1, 2002, to December 31, 2020, were converted (CDM version 5.0 of the Observational Health Data Sciences and Informatics database framework); these were termed “K-CDM data”. The K-CDM data of each hospital were stored separately in Microsoft’s structured query language (SQL) server; data of interest were queried and extracted using SQL.

Patients with CKD were defined when the participants had at least two laboratory findings apart more than 90 days prior to evaluation of estimated glomerular filtration rate (eGFR) < 60 mL/min/1.73 m^2^, calculated using creatinine based on the CKD-EPI equation (11), and the follow-up period was measured from the latter. Patients prescribed statins > 90 days after a diagnosis of CKD were defined as the treatment group. The Institutional Review Board of KUGH reviewed the study protocol (2020GR0255). Because the data were anonymized prior to analysis, patient consent was not required. The algorithm for patient enrollment is summarized in **Supplementary Figure 1**.

### Outcome Assessment

Data regarding the dates and causes of mortality were extracted from death certificates registered in electronic health records for the period of 2002–2020. All-cause mortality was analyzed for all cases; cardiovascular mortality was also analyzed, using ICD codes of ischemic heart disease, congestive heart failure, cerebrovascular disease, and peripheral vascular disease.

### Other Definitions

Hypertension was defined using the following ICD-10 codes: I10.0–I.15.0. Other comorbidities were defined using the following ICD-10 codes: diabetes mellitus (E10–E14), ischemic heart disease (I21.x, I22.x, I25.2, I20.x, I23.x, I24.x, and I25.x), congestive heart failure (I09.9, I11.0, I13.0, I13.2, I25.5, I42.0, I42.5–I42.9, I43.x, I50.x, and P29.0), cerebrovascular disease (G45.x, G46.x, H34.0, and I60.x–I69.x), and cancer (C00.x–C26.x, C30.x–C34.x, C37.x–C41.x, C43.x, C45.x–C58.x, C60.x–C76.x, C81.x–C85.x, C88.x, and C90.x–C97.x). Smoking status and alcohol consumption were defined using the concept IDs in the OBSERVATION table of the OMOP-CDM database. Detailed information regarding concept IDs used in the analyses is summarized in **Supplementary Table 1**.

Among the covariates, the laboratory finding, smoking status and alcohol consumption used data at the closest point to the index date performed within 1 year before the definition of CKD. Except for the Charlson comorbidity index (CCI), any other comorbidities was diagnosed at any time during the 1-year screening period prior to enrollment. For CCI, time of measurement was 1 year prior to the date of the index where CKD was defined.

### Sensitivity Analysis Using Korea National Health Insurance (KNHI) Claims Database

Analyzed results based on K-CDM data were confirmed with s analysis based on the Korea National Health Insurance (KNHI) claims database of the Health Insurance Review and Assessment Service. Dialysis was included according to the codes V001 and V003 between January 2003 and December 2015; CKD was diagnosed between January 2003 and December 2013 by using ICD codes N18.x and N19.x. The KNHI database contains information concerning all medical claims of approximately 50 million Koreans. It is based on the data submitted by healthcare providers regarding their medical procedures for the Health Insurance Review and Assessment Service review process, which is an essential step for the reimbursement of medical costs in Korea. It also contains the results of regular medical examinations through the KNHI examination program, performed every other year for the entire Korean population > 40 years of age who choose to voluntarily undergo the examination. Participants with ICD-10 codes N18 and N19 were defined as CKD patients; patients undergoing dialysis treatment were defined with a special code assigned to maintenance dialysis procedures > 1 months (V001, hemodialysis; V003, peritoneal dialysis). Statin treatment was defined as the prescription of any statin > 90 days after the registration of a CKD diagnosis or the initiation of dialysis. Data concerning comorbid conditions were extracted using the same ICD-10 codes as the analysis of the K-CDM database. Data concerning all-cause mortality were defined as the termination of the medical claim in the KNHI database. The study protocol was reviewed by the Institutional Review Board of KUGH (2021GR0353). The algorithm for patient enrollment and the characteristics of included patients are summarized in **Supplementary Figure 2** and **Supplementary Table 2**.

### Statistical Analyses

Data were expressed as means ± standard deviations for continuous variables and as n (%) for categorical variables. The chi-squared test and analysis of variance were used to compare categorical and continuous variables, respectively. Differences in age, duration of follow-up, CCI, and laboratory parameters were compared using two-sample t-tests. Primary outcome was all-cause mortality, and Cox regression analysis was conducted after assessing violations of the proportional hazards assumption. Multiple clinical variables including age, sex, diabetes, hypertension, congestive heart failure, cerebrovascular disease, ischemic heart disease cancer, eGFR, hemoglobin, albumin and total cholesterol were used for multivariable analyses in K-CDM cohort. For comparison of cardiovascular mortality, we conducted competing risk analysis as well as conventional Cox regression. In confirmatory analysis using KNHI cohort, multivariable Cox regression analyses were performed using adjustment variables including age, sex, diabetes, hypertension, congestive heart failure, cerebrovascular disease, ischemic heart disease and cancer. Statistical analyses were conducted using all available data without imputation of missing values. All tests were two-sided at a significance level of 0.05. All statistical analyses were performed using Statistical Analysis Software, version 9.4 (SAS Institute, Cary, NC, USA).

## Results

### Demographic and Clinical Characteristics

Patients in each hospital who had serum creatinine levels measured from February 2002 to December 2020 were screened through the common data model (K-CDM). Of 648,353 screened patients in KUGH, and 590,312 screened patients in KUAH, the clinical data from 44,431 and 64,165 patients, respectively, were assessed after application of the exclusion criteria (**Supplementary Figure 1**).

The baseline characteristics of the patient datasets in each hospital are listed in **Table 1**. Among patients from KUGH and KUAH, the numbers of statin users were 7,467 (16.80%) and 13,212 (20.59%), respectively. Comparing the baseline demographic characteristics between statin users and non-users, statin users were younger, with a lower proportion of men, higher alcohol and tobacco consumption, and higher prevalences of major comorbidities (e.g., hypertension, diabetes mellitus, ischemic heart disease, congestive heart failure, and cerebrovascular disease), which led to higher CCI. These differences in baseline characteristics were similarly observed in both centers. The mean eGFR values among all patients in KUGH and KUAH were 43.7 ± 15.4 and 46.5 ± 13.8 mL/min/1.73 m^2^, respectively. In baseline laboratory tests, statin users in both centers had a lower eGFR, as well as higher total and LDL cholesterol levels (**Table 1**).

**Table 1:** Baseline characteristics of the study participants in two regional hospitals from K-CDM.

Variables	Korea University Guro Hospital			Korea University Anam Hospital		
	Total	Statin non-users	Statin users	Total	Statin non-users	Statin users
	(N = 44431)	(N = 36964)	(N = 7467)	(N = 64165)	(N = 50953)	(N = 13212)
Age, years – mean (SD)	67.6 ± 13.8	68.1 ± 13.9	65.2 ± 12.9	68.1 ± 13.2	68.7 ± 13.3	65.7 ± 12.5
Male sex, n (%)	25968 (58.45)	21779 (58.92)	4189 (56.1)	37470 (58.4)	29875 (58.63)	7595 (57.49)
Follow-up period, years – mean (SD)	8.9 ± 5.8	8.8 ± 5.8	9.5 ± 5.6	9.3 ± 5.9	9.2 ± 5.9	9.8 ± 5.8
Alcohol consumption, n (%)	6,515 (14.66)	5,234 (14.16)	1,281 (17.16)	10631 (16.57)	7966 (15.63)	2665 (20.17)
Smoking tabacco, n (%)	5,153 (11.6)	4,147 (11.22)	1,006 (13.47)	7443 (11.6)	5609 (11.01)	1834 (13.88)
Hypertension, n (%)	23,842 (53.66)	17,214 (46.57)	6,628 (88.76)	34065 (53.09)	22523 (44.2)	11542 (87.36)
Diabetes mellitus, n (%)	35,135 (79.08)	27,812 (75.24)	7,323 (98.07)	49913 (77.79)	37099 (72.81)	12814 (96.99)
Ischemic heart disease, n (%)	5,256 (11.83)	2,580 (6.98)	2,676 (35.84)	7902 (12.32)	3553 (6.97)	4349 (32.92)
Congestive heart failure, n (%)	3,331 (7.5)	2,065 (5.59)	1,266 (16.95)	5003 (7.8)	2878 (5.65)	2125 (16.08)
Cerebrovascular disease, n (%)	5,997 (13.5)	3,892 (10.53)	2,105 (28.19)	9277 (14.46)	5535 (10.86)	3742 (28.32)
Cancer, n (%)	12,863 (28.95)	11,336 (30.67)	1,527 (20.45)	16802 (26.19)	13967 (27.41)	2835 (21.46)
Liver disease, n (%)	4,820 (10.85)	4,147 (11.22)	673 (9.01)	6096 (9.5)	4805 (9.43)	1291 (9.77)
Chronic obstructive pulmonary disease, n (%)	3,129 (7.04)	2,367 (6.4)	762 (10.2)	3519 (5.48)	2496 (4.9)	1023 (7.74)
Charlson comorbidity index – mean (SD)	3.9 ± 2.2	3.8 ± 2.2	4.5 ± 2.1	3.8 ± 2.1	3.6 ± 2.0	4.4 ± 2.1
Hemoglobin, g/dL – mean (SD)	12.3 ± 2.4	12.2 ± 2.4	12.5 ± 2.3	12.7 ± 2.3	12.6 ± 2.4	13.0 ± 2.2
Creatinine, mg/dL – mean (SD)	2.0 ± 1.9	2.0 ± 1.9	2.1 ± 2.2	1.8 ± 1.67	1.7 ± 1.6	1.9 ± 2.1
eGFR, ml/min/1.73m^2^ – mean (SD)	43.7 ± 15.4	43.8 ± 15.2	43.2 ± 16.0	46.5 ± 13.8	46.6 ± 13.7	45.9 ± 14.5
Total cholesterol, mg/dL – mean (SD)	168.3 ± 50.5	165.5 ± 49.6	182.1 ± 52.3	171.4 ± 49.3	167.5 ± 48.0	183.6 ± 51.4
LDL cholesterol, mg/dL – mean (SD)	97.9 ± 41.3	93.8 ± 39.6	108.3 ± 43.6	99.6 ± 37.7	95.3 ± 35.9	107.8 ± 39.6
Triglyceride, mg/dL – mean (SD)	138.0 ± 105.2	129.8 ± 99.5	161.5 ± 116.7	145.7 ± 116.4	137.0 ± 103.9	165.2 ± 138.5
HDL cholesterol, mg/dL – mean (SD)	42.5 ± 16.3	41.9 ± 16.8	44.3 ± 14.7	43.5 ± 14.7	43.1 ± 15.2	44.6 ± 13.6
C-reactive protein, mg/L – mean (SD)	43.0 ± 70.7	46.1 ± 72.8	32.1 ± 61.5	38.0 ± 69.6	42.8 ± 73.2	24.0 ± 56.0
Albumin, g/dL – mean (SD)	3.86 ± 0.6	3.84 ± 0.61	3.99 ± 0.54	3.95 ± 0.67	3.91 ± 0.68	4.1 ± 0.59

eGFR, estimated glomerular filtration rate; LDL, low density lipoprotein; HDL, high density lipoprotein.

### Comparison of All-Cause and Cardiovascular Mortality Between Statin Users and Non-Users

During the follow-up period for 8.9 ± 5.8 years and 9.5 ± 5.6 years in KUGH and KUAH participant, 8,275 (18.62%) and 9,604 (14.97%) died. In both centers, statin users showed lower hazard ratio for all-cause mortality as well as cardiovascular mortality compared to statin non-users (**Table 2**). In KUGH, statin users were associated with 67% and 78% lower risks for all-cause mortality and CV mortality, respectively. In parallel, statin users in KUAH showed 61% and 78% lower risks for all-cause mortality and CV mortality, respectively. Additionally, CV mortality was analysed by competing risk analysis, and the results were similar to conventional Cox regression (**Supplementary Table 3**).

**Table 2:** Risk of all-cause mortality and cardiovascular mortality according to statin use in dataset from K-CDM of regional hospitals.

	Unadjusted HR (95% CI)	Adjusted HR (95% CI)^*^
***All-cause mortality***		
Statin users (KUGH)	0.36 (0.34-0.40)	0.33 (0.30-0.36)
Statin users (KUAH)	0.41 (0.39-0.44)	0.39 (0.36-0.42)
***Cardiovascular mortality***		
Statin users (KUGH)	0.59 (0.48-0.72)	0.22 (0.18-0.27)
Statin users (KUGH)	0.50 (0.42-0.59)	0.22 (0.19-0.27)

*Adjusted for age, sex, Congestive heart failure, Cerebrovascular disease, cancer, eGFR, Hemoglobin, Albumin, Total cholesterol.

HR, hazard ratio; CI, confidence interval; KUGH, Korea University Guro Hospital; KUAH, Korea University Anam Hospital.

### *Post-Hoc* Subgroup Analyses

**Figure 1:**
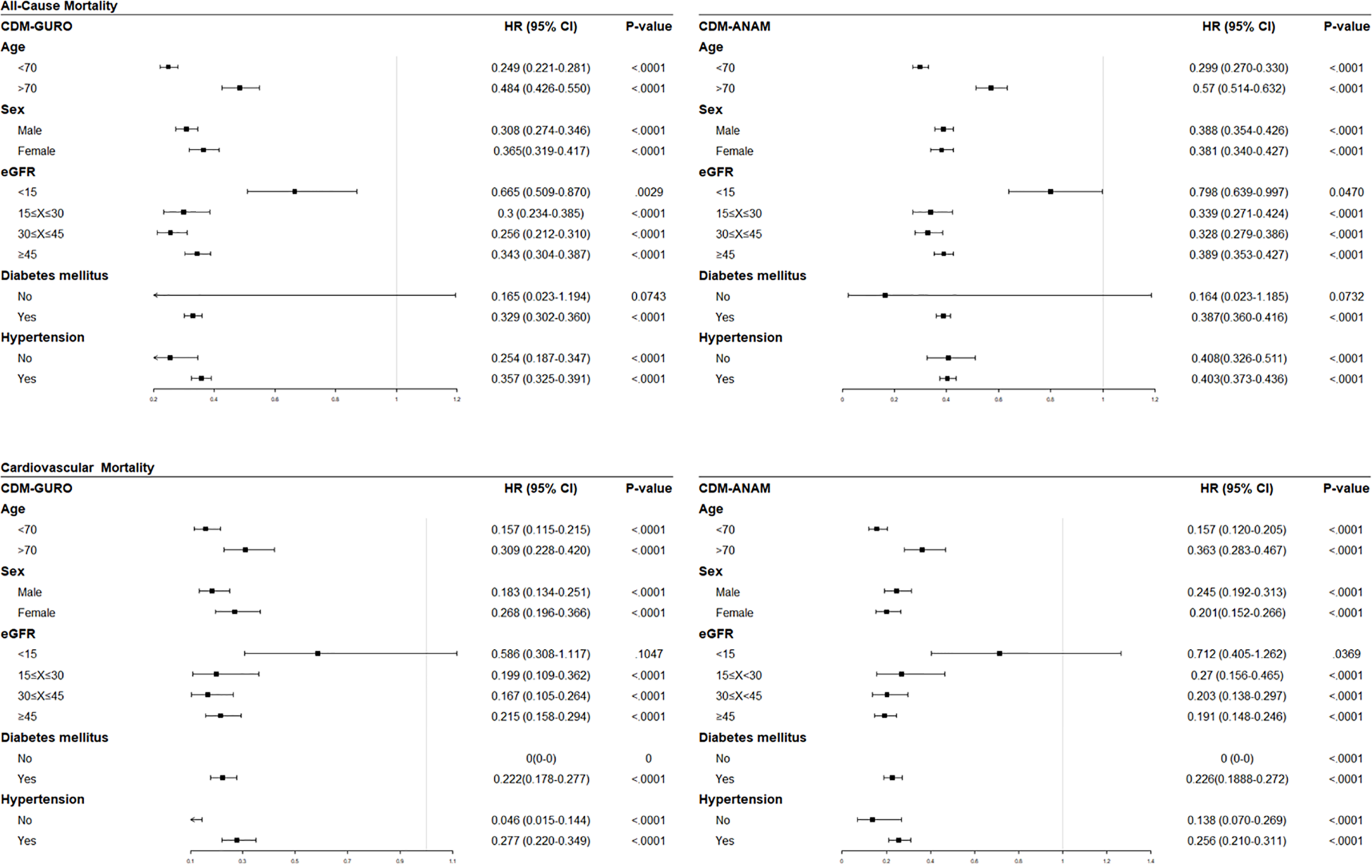
Forest plots of subgroup analyses showing effect of statins on all-cause mortality (upper row) and cardiovascular mortality (lower row) analyzed the data from K-CDM. eGFR, estimated glomerular filtration rate; HR, hazard ratio; CI, confidence interval.

Based on significant results in the adjusted multivariable analysis, subgroup analyses were performed to assess changes in risk according to separate clinical variables (**Figure 1**). In both centers, the risk of all-cause mortality was consistently reduced in statin users regardless of age, sex, renal function, and the presence of diabetes or hypertension. In a specific subgroup with eGFR under 15 ml/min/1.73m^2^, which is eligible population for dialysis, statin users were found to be associated with reduced risk of all-cause mortality but not with cardiovascular mortality.

In accordance with the recent Kidney Disease: Improving Global Outcomes lipid management guidelines for CKD (5), we further analysed the risk difference after statin use in low-risk young patients (not currently recommended to receive statin treatment) and in other patients. We found that the risks for all-cause mortality and cardiovascular mortality were significantly lower among statin users compared to non-users, both in low-risk patients < 50 years of age and in other patients (**Table 3**).

**Table 3:** All-cause and cardiovascular mortality in patients with kidney dysfunction following statin usage, analyzed in K-CDM of regional hospitals.

	KUGH		KUAH	
	Number of events/total patients	Adjusted HR^*^ (95% CI)	Number of events/total patients	Adjusted HR^*^ (95% CI)
***All-cause mortality***				
Statin users in age ≥50	7255/38013	0.39 (0.36-0.42)	8520/48694	0.48 (0.45-0.52)
Statin users in age <50, with one or more risk factors**	695/1946	0.12 (0.08-0.18)	663/2042	0.12 (0.04-0.18)
Statin users in age <50 without risk factors**	325/2756	0.13 (0.07-0.25)	421/3040	0.28 (0.19-0.41)
***Cardiovascular mortality***				
Statin users in age ≥50	787/38013	0.43 (0.35-0.54)	1122/48694	0.51 (0.42-0.61)
Statin users in age <50, with one or more risk factors**	74/1946	0.03 (0.004-0.20)	82/2130	0.10 (0.04-0.29)
Statin users in age <50 without risk factors**	30/2756	0.22 (0.05-0.98)	62/3040	0.13 (0.04-0.42)

*All hazard ratios were obtained after adjusting for age, sex, hypertension, congestive heart failure, cancer, eGFR, hemoglobin, albumin, total cholesterol.

**Risk factors were ischemic heart disease, cerebrovascular disease or diabetes mellitus.

KUGH, Korea University Guro Hospital; KUAH, Korea University Anam Hospital; HR, hazard ratio; CI, confidence interval; NA, not available.

### Confirmatory Analysis Based on Sample Cohort Database Of Korean National Health Insurance Services

Confirmatory analysis was performed to determine the reproducibility of the results from K-CDM; the claims data for 4,114 individuals with CKD diagnosed between January 2003 and December 2013 according to ICD codes from a sample cohort database from KNHI were evaluated for analysis. The baseline characteristics of non-dialysis CKD, hemodialysis, and peritoneal dialysis patients are listed in **Supplementary Table 2**. When comparing all-cause mortality risk between statin users and non-users in dialysis and non-dialysis CKD patients, statin users in non-dialysis patients were associated with 59% lower risk compared to non-users. And statin users in hemodialysis and peritoneal dialysis patients showed 36% lower risks compared to non-users, respectively (**Table 4**).

**Table 4:** All-cause mortality in chronic kidney disease patients following statin usage, analyzed in sample data cohort from KNHI.

	Non-dialysis CKD N of Statin users/Total = 984/4114 (23.9%)		Hemodialysis N of Statin users/Total = 5416/37998 (14.3%)		Peritoneal dialysis N of Statin users/Total = 977/4866 (20.1%)	
	Event N (%)	Adjusted HR^*^ (95% CI)	Event N (%)	Adjusted HR^*^ (95% CI)	Event N (%)	Adjusted HR^*^ (95% CI)
Statin users (Sample cohort from KNHI)	129 (13.1%)	0.41 (0.34-0.50)	1293 (23.9%)	0.39 (0.35-0.43)	175 (17.9%)	0.34 (0.25-0.46)

*For non-dialysis CKD, All hazard ratios were obtained after adjusting for age, sex, cancer, cerebrovascular disease, congestive heart failure, diabetes mellitus, hypertension, ischemic heart disease.

For hemodialysis, peritoneal dialysis, All hazard ratios were obtained after adjusting for age, sex, cerebrovascular disease, congestive heart failure, diabetes mellitus, hypertension, myocardial infarction, any malignant tumors, hemoglobin, creatinine.

CKD, chronic kidney disease; HR, hazard ratio; CI, confidence interval; NHIS, National Health Insurance System.

## Discussion

In this observational study using two cohorts obtained from OMOP-CDM and KNHI databases, statin use at various stages of CKD was associated with lower cardiovascular mortality and all-cause mortality. In a *post-hoc* subgroup analysis, the benefits of statin use were still observed in dialysis-dependent CKD participants, who showed inconsistent results in previous studies. The present study provided real-world evidence for statin use in various CKD populations

Considerable proportions of the high morbidity and mortality rates among CKD patients are associated with CVD. CVD is the most common estimated cause of death in advanced CKD patients with an eGFR < 60 ml/min/1.73m^2^; the likelihood of cardiovascular mortality increases with decreasing eGFR (12). Among causes of cardiovascular mortality, ischemic heart disease is the most common, constituting > 50% of cardiovascular deaths (12). A notable pathophysiological contribution to the high cardiovascular mortality in CKD patients is lipoprotein alteration; a specific lipid profile alteration known as “uremic dyslipidemia” occurs in CKD patients, involving normal LDL cholesterol, low high-density lipoprotein cholesterol, and high triglyceride levels (13). In addition to changes in lipoprotein composition, an increase in apolipoprotein carbamylation and functional changes in lipoproteins can lead to proinflammatory cytokine production (14, 15). Therefore, the management of uremic dyslipidemia may be a promising therapeutic target to prevent CKD-associated CVD.

The beneficial effect of statin treatment in CKD patients has been proven in *post hoc* analyses of various clinical trials in the general population (16–19), as well as a randomized controlled trial in a CKD population. The SHARP study showed that simvastatin/ezetimibe may reduce the composite outcome in a CKD population by 17% (20). The results of our study were consistent with the previous findings. However, in dialysis patients, a specific subpopulation of CKD patients, the efficacy of statins has not yet been proven in randomized controlled trials (21, 22). Neither the 4D study, which prescribed atorvastatin for diabetic dialysis patients, nor the AURORA study, which prescribed rosuvastatin for dialysis patients aged 50–80 years, could prove a protective effect of statin treatment in a dialysis population (21, 22). However, based on the consistent protective effects of statins in previous large-scale observational studies of dialysis populations (23–25), and in our study using two databases, the potential desirable effect of statins in this specific population warrants further investigation.

The cause of these conflicting findings is unclear, but several possibilities should be considered (26). First, two randomized controlled trials reported high drop-out and drop-in rates. In the 4D study, 17% of the statin arm patients discontinued treatment, while 15% of the placebo group received non-study statins (21). In the AURORA study, 50% of the patients dropped out of both treatment groups (22). Additionally, the 4D study excluded patients with an LDL cholesterol level ≥ 190 mg/dL (21); the AURORA study excluded patients who were administered statins within 6 months prior to study inclusion (22), suggesting the possible exclusion of patients who might experience greater benefits from statins in both studies. Furthermore, differences in ethnicity among study populations should be considered. The pharmacokinetics and pharmacodynamics of drugs often show large differences according to ethnicity; several studies in Korean patients showed higher LDL cholesterol reduction by statins, compared with studies involving other ethnicities (27–34).

This study revealed better survival with statin use in a low-risk CKD population under 50 years of age, as well as in a dialysis population. According to the Kidney Disease: Improving Global Outcomes guidelines, which are widely adopted for the management of CKD patients, statins should only be considered in patients > 50 years of age, or in patients < 50 years of age with a high risk of CVD (5). However, the significant results in young low-risk CKD patients in our study suggest an underestimated cardiovascular risk in young CKD patients and the universal effect of statins in this population. In a recent study, cardiovascular mortality in young end-stage renal disease patients in their 20s was distinct from the risk in children, and more similar to the risk in older adults; it was 143–500-fold greater than in the age-matched general population (35). Therefore, the assessment of cardiovascular risk and the reconsideration of statin use in young CKD patients may be necessary.

A notable study strength was the reproducibility of similar results validated by two different large-scale databases. Using the established common database model between hospitals, statistical analysis was performed using identically mapped variables that included laboratory results. The validation analysis was performed using a sample cohort database, randomly extracted from national insurance data to resolve regional differences; these regional differences constitute a disadvantage of using hospital datasets. Because the analyses in this study used two databases, the universality and reliability of the results was maintained.

However, there are several limitations also exist. First, the present observational cohort study may have been biased and could not clearly suggest a causal relationship; furthermore, specific laboratory variables (e.g., LDL cholesterol and C-reactive protein) could not be included because they exhibited high rates of missing values. The adherence to statin use was not evaluated nor if additional statins were started in non-user group during the rest of the follow up duration. A survival bias could have been introduced because deaths prior to 90 days of statin use were excluded. And defining a statin user as an arbitrary period of 90 days could also lead to a bias. Not all potential variables affecting all-cause and CV mortality were adjusted which may create residual bias. Additionally, we were unable to adjust lipid profiles in the validation analysis using the sample cohort because laboratory results were not available in the claims database.

CKD patients have complex and diverse inflammatory profiles; this intra-disease heterogeneity makes it difficult to define the population of statin users among CKD patients. Our study revealed consistent results in patients with CKD of various stages, confirming the association between statin use and reduced mortality found in previous studies; we also found beneficial effects in dialysis patients and young CKD patients, although such effects remain controversial. A prospective study of various subpopulations is needed to help determine appropriate lipid management in CKD patients.

## Data Availability Statement

The raw data supporting the conclusions of this article will be made available by the authors, without undue reservation.

## Ethics Statement

The studies involving human participants were reviewed and approved by Institutional Review Board of Korea University Guro Hospital. Written informed consent for participation was not required for this study in accordance with the national legislation and the institutional requirements.

## Author Contributions

GJ-K conceived of the presented idea. JK and YC carried out the analysis and interpretation of data. JK, YC, and GJ-K wrote the manuscript with support from SA and YK. All authors have read and agreed to the published version of the manuscript.

## Funding

This work was supported by Institute of Korea Health Industry Development Institute (KHIDI) grant funded by the Korea government (Ministry of Health and Welfare, MOHW) (No. HI19C0806, the development of information management system and security tools to avoid the invasion threat of CDM information security based on laws for utilization of distributed research network).

## Conflict of Interest

The authors declare that the research was conducted in the absence of any commercial or financial relationships that could be construed as a potential conflict of interest.

## Publisher’s Note

All claims expressed in this article are solely those of the authors and do not necessarily represent those of their affiliated organizations, or those of the publisher, the editors and the reviewers. Any product that may be evaluated in this article, or claim that may be made by its manufacturer, is not guaranteed or endorsed by the publisher.
