# Supplementary material for: The Effect of Statins on Mortality of Patients With Chronic Kidney Disease Based on Data of the Observational Medical Outcomes Partnership Common Data Model (OMOP-CDM) and Korea National Health Insurance Claims Database
Source: Front Nephrol. 2022 Feb 2;1:821585. doi: 10.3389/fneph.2021.821585 (PMC10479676; doi:10.3389/fneph.2021.821585)
Supplement: Supplementary file 1 [file DataSheet_1.docx]

Supplementary Material

# Supplementary Figures and Tables

**Supplementary Figure 1.** Study schema based on K-CDM

1. KUGH


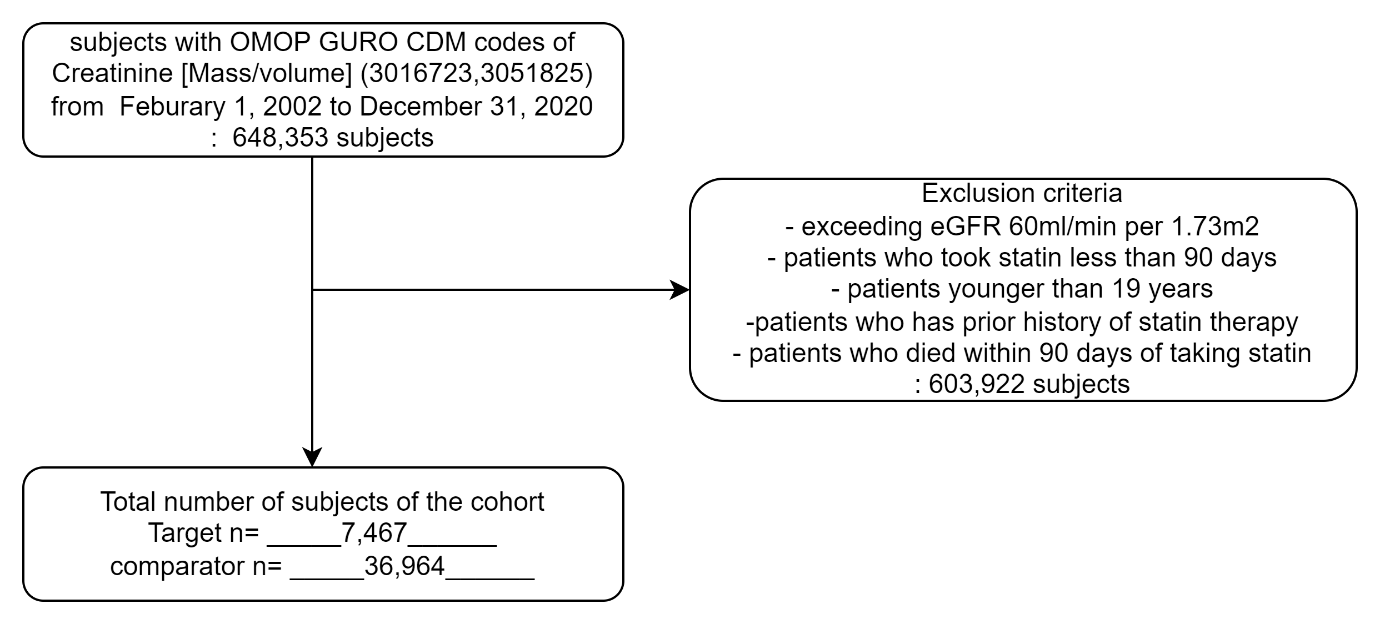


1. KUAH


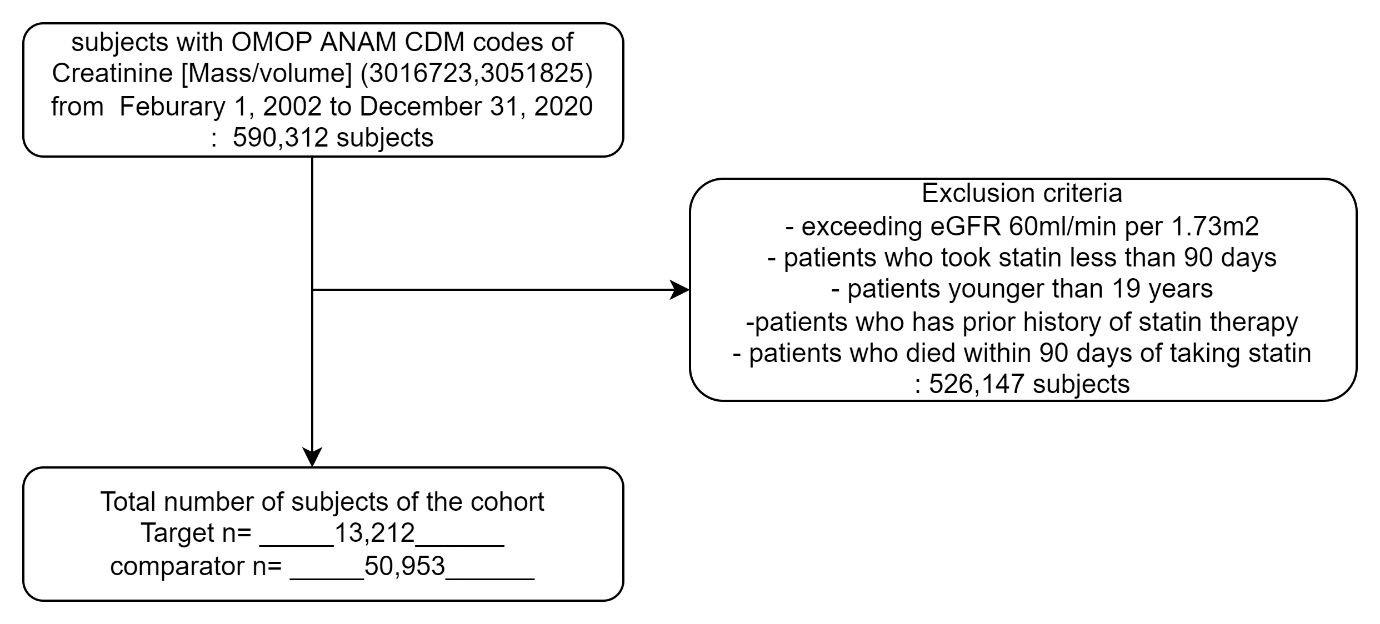


**Supplementary Table 1**. Lists of concept id used in the analysis

| **ICD-10TH CONDITION** | Concept ID |
| --- | --- |
| Diabetes mellitus | E10.xx - 14.xx |
| Hypertension | I10.xx - 15.xx |
| Myocardial infarction | I21.x, I22.x, I25.2 |
| Congestive heart failure | I09.9, I11.0, I13.0, I13.2, I25.5, I42.0, I42.5 - I42.9, I43.x, I50.x, P29.0 |
| Peripheral vascular disease | I70.x, I71.x, I73.1, I73.8, I73.9, I77.1, I79.0, I79.2, K55.1, K55.8, K55.9, Z95.8, Z95.9 |
| Cerebrovascular disease | G45.x, G46.x, H34.0, I60.x - I69.x |
| Dementia | F00.x - F03.x, F05.1, G30.x, G31.1 |
| Chronic pulmonary disease | I27.8, I27.9, J40.x - J47.x, J60.x - J67.x, J68.4, J70.1, J70.3 |
| Rheumatic disease | M05.x, M06.x, M31.5, M32.x - M34.x, M35.1, M35.3, M36.0 |
| Peptic ulcer disease | K25.x - K28.x |
| Mild liver disease | B18.x, K70.0 - K70.3, K70.9, K71.3 - K71.5, K71.7, K73.x, K74.x, K76.0, K76.2 - K76.4, K76.8, K76.9, Z94.4 |
| Diabetes without chronic complication | E10.0, E10.1, E10.6, E10.8, E10.9, E11.0, E11.1, E11.6, E11.8, E11.9, E12.0, E12.1, E12.6, E12.8, E12.9, E13.0, E13.1, E13.6, E13.8, E13.9, E14.0, E14.1, E14.6, E14.8, E14.9 |
| Diabetes with chronic complication | E10.2 - E10.5, E10.7, E11.2 - E11.5, E11.7, E12.2 - E12.5, E12.7, E13.2 - E13.5, E13.7, E14.2 - E14.5, E14.7 |
| Hemiplegia or paraplegia | G04.1, G11.4, G80.1, G80.2, G81.x, G82.x, G83.0 - G83.4, G83.9 |
| Renal disease | I12.0, I13.1, N03.2 - N03.7, N05.2 - N05.7, N18.x, N19.x, N25.0, Z49.0 - Z49.2, Z94.0, Z99.2. |
| Any malignancy, including lymphoma and leukaemia, except malignant neoplasm of skin: | C00.x - C26.x, C30.x - C34.x, C37.x - C41.x, C43.x, C45.x - C58.x, C60.x - C76.x, C81.x - C85.x, C88.x, C90.x - C97.x |
| Moderate or severe liver disease | I85.0, I85.9, I86.4, I98.2, K70.4, K71.1, K72.1, K72.9, K76.5, K76.6, K76.7 |
| Metastatic solid tumor | C77.x - C80.x |
| AIDS/HIV | B20.x - B22.x, B24.x |
| **OMOP DRUG/VARS** | Concept ID |
| Hemoglobin | 3000963 |
| Total cholesterol | 3027114 |
| Triglyceride | 3022192 |
| HDL cholesterol | 3007070 |
| LDL cholesterol | 3028437 |
| Alcohol consumption | 4052351 |
| Smoking tobacco | 4041306 |
| Statins | 2009678, 2009679, 2011379, 2011381, 2011383, 2029667, 2029668, 2032232, 2032233, 2034494, 2034495, 2034528, 2034568, 2067217, 2067219, 2067224, 19064528, 40230687, 4023068, 40231109, 40830052, 40850083, 40957798, 41005943, 41112138, 41208244, 41239186, 41414546, 41425705, 42899008, 42924483, 42924515, 42924548, 42962628, 42962726, 42962826, 42972679 |
| Diabetes mellitus | 19021312, 1529352, 40164943, 43013911, 43013924, 42953740, 42953698, 42953917, 42953818, 19122366, 19122367, 19122368, 1545997, 40164929, 40164939, 19123592, 40164925, 40164885, 40164916, 40164919, 1594976, 42960765, 42960762, 42960759, 44785831, 45775456, 45775620, 42961331, 45774754, 45774893, 46287680, 46287689, 46287408, 46287686, 21130197, 21091002, 19077682, 19059797, 19101729, 21133671, 42960642, 42960645, 42960648, 42961500, 40165997, 40166002, 42942992, 1597772, 1597761, 1597758, 1597773, 19006931, 21169719, 43267262, 21081251, 42962855, 40164891, 40164922, 36887702, 42708168, 42708172, 42708176, 40231394, 40231402, 42960773, 40239218, 42708086, 42708088, 42708090, 40164897, 19106521, 40164946, 40164894, 42962884, 1502829, 19107111, 19107110, 42960653, 43013915, 43013918, 43013896, 43013899, 1525221, 19079293, 1547508, 19079465, 19023425, 19023424, 19023426, 19125041, 19125049, 42961319, 42961322, 42961325, 40166037, 40166041, 42960599, 42960587, 42960590, 42960593, 42961179, 42961189, 42961170, 42961173, 19129179, 19112791, 42902468, 42921644, 42922767, 42922959, 42921783, 42922264, 46234234, 19078603, 19058398, 46233974, 42902587, 42902945, 46233971, 19078552, 46234239, 19078555, 19135264, 43275300, 42902821, 40169222, 19078559, 42902742, 41349138, 41349636, 43518492, 41349148, 41348682, 41348912, 1596972, 46234050, 42902356, 46234047, 46234237, 43297029, 19078558, 42921712, 42921721, 46221558, 35602725, 42961487, 42961484, 42961490, 42961494, 42969162, 42969165, 42969168, 42902742, 19078559, 46221558, 42921721, 43518492, 41348912, 41348682, 41349148, 35602725, 42921712, 1531601 ,42902356, 42902587, 1513876, 42902945, 1516976, 42902821, 43297029, 42921644, 43275300, 41348912, 41348682, 41349148, 1544838, 19112791, 42902468, 46234050, 19078558, 42921712, 46234047, 19058398, 1513876, 42902587, 46233971, 42902945 |
| hypertension | 974447, 974473, 974474, 974642, 974702, 1305450, 1308851, 1308874, 1309071, 1318859, 1318860, 1319942, 1319943, 1321637, 1326020, 1328585, 1328689, 1332419, 1332494, 1332495, 1332497, 1332499, 1332525, 1332527, 1334461, 1334492, 1337070, 1337103, 1340161, 1351559, 1351583, 1351587, 1353780, 1353818, 1353820, 1592858, 19011548, 19011549, 19015804, 19017656, 19019309, 19020063, 19022241, 19022242, 19022948, 19022949, 19023453, 19023454, 19028935, 19028936, 19050220, 19058101, 19073093, 19073094, 19074672, 19074673, 19076924, 19078080, 19078101, 19080128, 19081025, 19096677, 19096678, 19096740, 19096752, 19101748, 19101750, 19101751, 19101807, 19102170, 19102171, 19102491, 19106542, 19106543, 19106593, 19106594, 19107180, 19112606, 19112979, 19112981, 19113063, 19121182, 19122209, 19127432, 19127433, 19127434, 19133612, 19133613, 19134566, 21030573, 21041304, 21056210, 21058150, 21070867, 21097731, 21141332, 21601761, 35604949, 35604953, 35604961, 36883910, 40069686, 40163142, 40163271, 40163275, 40163753, 40163760, 2064553, 2064498, 2064586, 40165762, 40165789, 40167202, 40167843, 40167849, 40167852, 40171661, 40171863, 40171884, 40171905, 40171917, 40174776, 40174811, 40184184, 40184187, 40184217, 40185276, 40185304, 40224166, 40224172, 40224175, 40235487, 40235491, 42707639, 42707641, 42801011, 42801015, 42925744, 42925749, 42925752, 42929931, 42929938, 42929951, 42930392, 42930395, 42932538, 42932541, 42932544, 42932547, 42938510, 42938513, 42938516, 42948648, 42948651, 42948654, 42950300, 42952826, 42955431, 42955514, 42959698, 42959787, 42959791, 42959999, 42960002, 42960005, 42960008, 42960013, 42960018, 42960027, 42960031, 42960035, 42960725, 42960735, 42960852, 42960860, 42960864, 42960868, 42961740, 42961744, 42963311, 42968981, 42968990, 42968999, 42969008, 42969022, 42969031, 42969040, 42969049, 42969058, 42969067, 42969082, 42969085, 42969088, 42969091, 42969094, 42969097, 42969123, 42969126, 42969129, 42969132, 42969135, 42969138, 42969141, 42969149, 42969152, 42969154, 42969157, 42972631, 42972634, 42972637, 42972640, 43275607, 43291777, 44507582, 46287343, 42950421, 42950354, 42950393, 42950866, 42950863, 42950869, 42969118, 42969112, 42969115, 42969109, 42969102, 42969106 |

**Supplementary Figure 2.** Algorism for the analysis of Korea National Health Insurance (KNHI) claims database

1. Chronic kidney disease


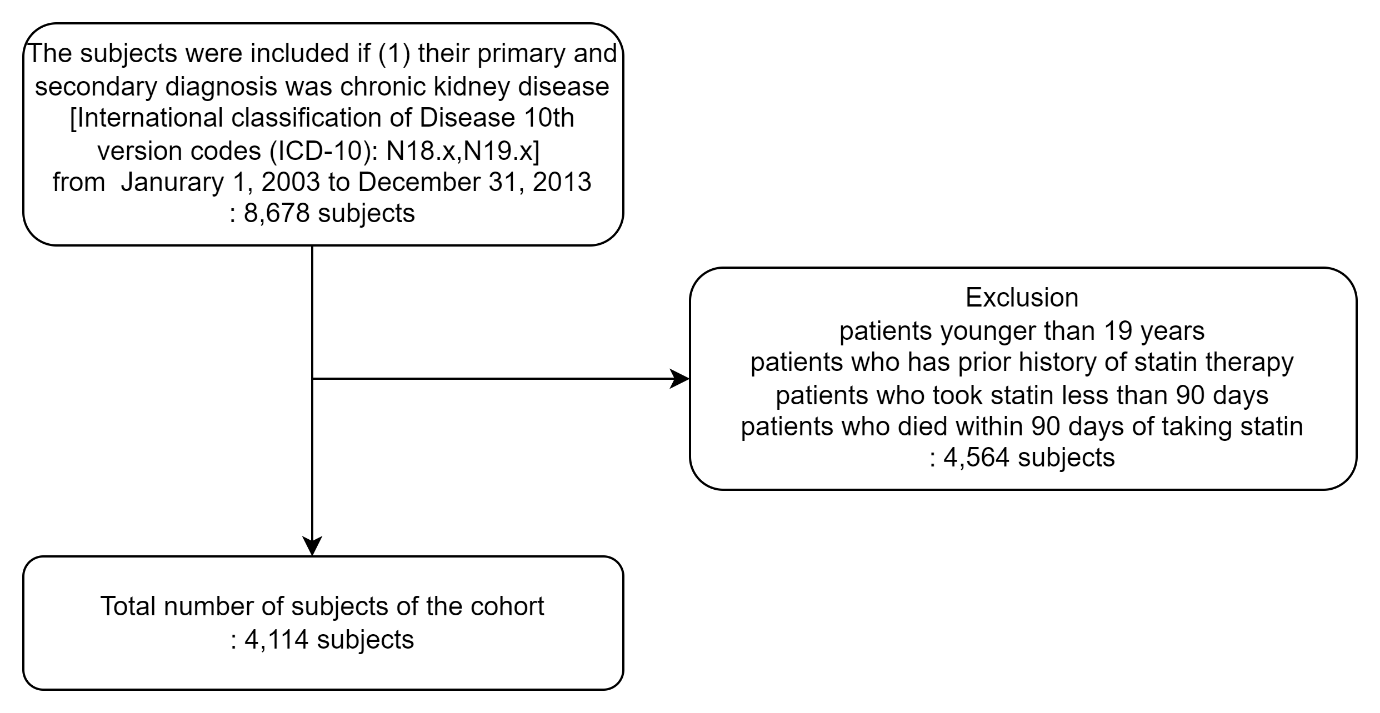


1. Hemodialysis / Peritoneal dialysis


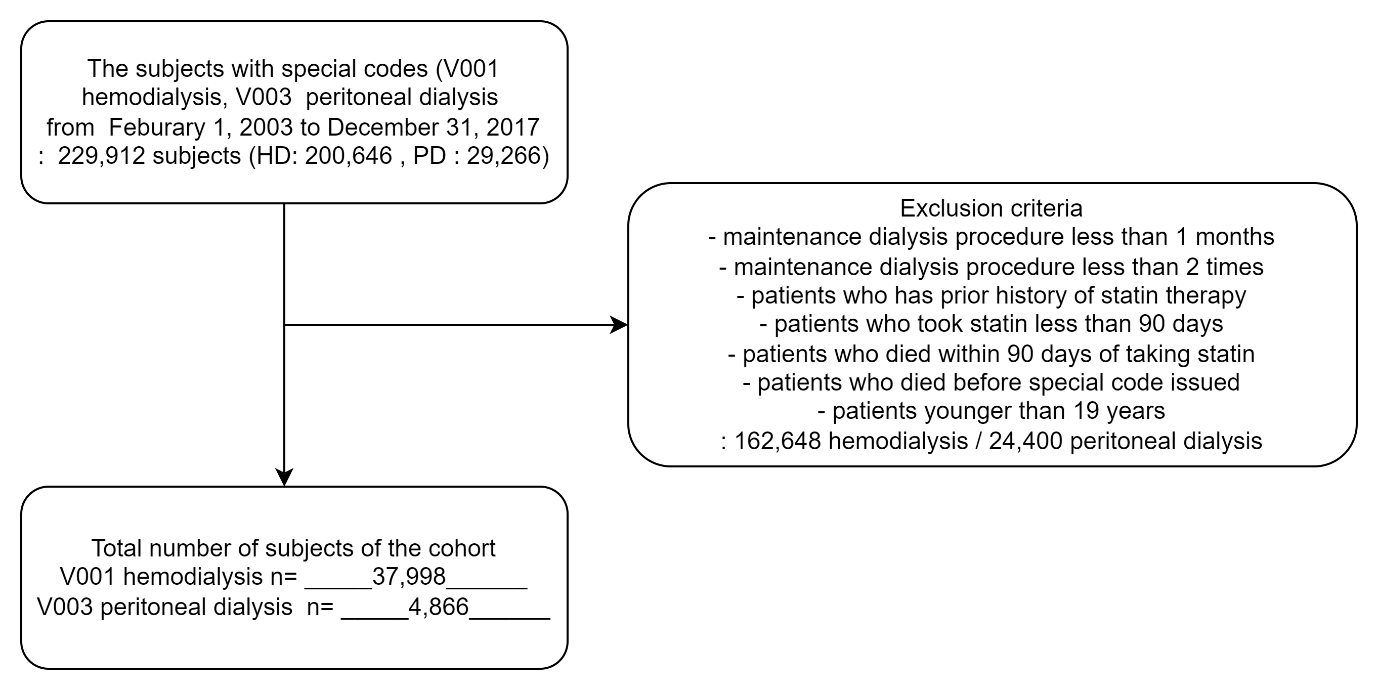


**Supplementary Table 2.** Baseline characteristics of CKD and dialysis patients extracted from Korea National Health Insurance (KNHI) claims database

1. Chronic kidney disease

| Variables | Total | Statin non-users | Statin | P-value |
| --- | --- | --- | --- | --- |
| Age, years | 64.69±10.93 | 65.52±11.08 | 62.05±10.01 | <0.0001 |
| Male sex, n (%) | 2,740 (66.6) | 2,147 (68.6) | 593 (60.3) | <0.0001 |
| Charson comorbidity index |  |  |  | <0.0001 |
| 0 | 1753 (42.61) | 1279 (40.86) | 474 (48.17) |  |
| 1 | 850 (20.66) | 628 (20.06) | 222 (22.56) |  |
| 2 | 718 (17.45) | 559 (17.86) | 159 (16.16) |  |
| 3 | 793 (19.28) | 664 (21.21) | 129 (13.11) |  |
| Diabetes mellitus, n (%) | 2456 (59.7) | 1765 (56.39) | 691 (70.22) | <0.0001 |
| Hypertension, n(%) | 3638 (88.43) | 2694 (86.07) | 944 (95.93) | <0.0001 |
| Ischemic heart disease, n(%) | 1459 (35.46) | 978 (31.25) | 481 (48.88) | <0.0001 |
| Congestive heart failure, n(%) | 973 (23.65) | 695 (22.2) | 278 (28.25) | <0.0001 |
| Cerebrovascular disease, n(%) | 1476 (35.88) | 1071 (34.22) | 405 (41.16) | <0.0001 |
| Cancer, n(%) | 1153 (28.03) | 934 (29.84) | 219 (22.26) | <0.0001 |
| COPD, n(%) | 2785 (67.7) | 2089 (66.74) | 696 (70.73) | 0.0196 |
| Liver disease, n(%) | 1842 (44.77) | 1380 (44.09) | 462 (46.95) | 0.1153 |

1. Hemodialysis / Peritoneal dialysis

| variables | **Hemodialysis** | | | | **Peritoneal dialysis** | | | |
| --- | --- | --- | --- | --- | --- | --- | --- | --- |
|  | Total | Statin non-users | Statin | P-value | Total | Statin non-users | Statin | P-value |
| Age, years | 58.4(±16.55) | 59.33(±16.72) | 52.82(±14.23) | <.0001 | 51.25(±18.28) | 52.02(±19.16) | 48.2(±13.79) | <.0001 |
| Male sex, n (%) | 23,823(62.7) | 20,658(63.40) | 3,165(58.44) | <.0001 | 3,000(61.65) | 2,497(64.21) | 503(51.48) | <.0001 |
| Alcohol consumption, n (%) | 1,845(9.33) | 1,586(9.78) | 259(7.30) | <.0001 | 171(7.12) | 136(7.76) | 35(5.38) | 0.0441 |
| Smoking tabacco, n (%) | 12,516(63.31) | 10,213(62.96) | 2,303(64.91) | 0.0887 | 1,583(65.96) | 1,120(63.96) | 463(71.34) | 0.0003 |
| Hypertension, n (%) | 22,439(59.05) | 18,406(56.49) | 4,033(74.46) | <.0001 | 3,046(62.6) | 2,270(58.37) | 776(79.43) | <.0001 |
| Diabetes mellitus, n (%) | 8,355(21.99) | 6,871(21.09) | 1,484(27.40) | <.0001 | 930(19.11) | 717(18.44) | 213(21.80) | 0.0168 |
| Charson comorbidity index | 3.55(±1.95) | 3.63(±1.99) | 3.09(±1.65) | <.0001 | 3.24(±1.84) | 3.34(±1.87) | 2.82(±1.62) | <.0001 |
| Body mass index, kg/m2 | 22.58(±3.32) | 22.31(±3.19) | 23.35(±3.54) | <.0001 | 23.16(±3.31) | 22.93(±3.21) | 23.61(±3.47) | <.0001 |
| Dialysis duration, days | 1334.67(±1197) | 1249.35(±1156.21) | 1573.43(±1274.58) | <.0001 | 1397.51(±1226.06) | 1326.35(±1207.09) | 1536.18(±1251.67) | 0.0008 |
| Creatinine, mg/dL | 6.01(±4.72) | 6.42(±4.61) | 4.89(±4.85) | <.0001 | 6.09(±6.45) | 6.78(±6.61) | 4.81(±5.95) | <.0001 |
| Hemoglobin, g/dL | 11.80(±1.85) | 11.66(±1.80) | 12.21(±1.92) | <.0001 | 11.88(2.04) | 11.75(±1.98) | 12.13(±2.12) | 0.0003 |
| Fasting blood glucose, mg/dL | 104.26(±39.05) | 103.89(±39.43) | 105.31(±37.95) | 0.0785 | 103.8(±36.48) | 103.4(±37.96) | 104.58(±33.45) | 0.5098 |
| Total cholesterol, mg/dL | 166.9(±42.04) | 163.28(±38.32) | 176.82(±49.54) | <.0001 | 177.05(±36.86) | 172.63(±35.02) | 181.56(±38.8) | <.0001 |
| HDL cholesterol, mg/dL | 51.17(±22.09) | 50.99(±22.2) | 51.66(±21.79) | 0.1695 | 51.73(±23.83) | 51.23(±27.53) | 52.65(±14.99) | 0.1874 |
| LDL cholesterol, mg/dL | 92.34(±41.33) | 90.5(±36.44) | 97.18(±51.76) | <.0001 | 100(±32.26) | 97.71(±29.84) | 104.12(±35.86) | 0.0004 |

**Supplementary Table 3.** Competing risk analysis for cardiovascular mortality in K-CDM database.

|  | Unadjusted HR (95% CI) | Adjusted HR (95% CI)^*^ |
| --- | --- | --- |
| Statin users (KUGH) | 0.66 (0.54-0.82) | 0.29 (0.24-0.37) |
| Statin users (KUGH) | 0.54(0.46-0.64) | 0.30 (0.25-0.36) |

* Adjusted for age, sex, Congestive heart failure, Cerebrovascular disease, cancer, eGFR, Hemoglobin, Albumin, Total cholesterol.

Abbreviations: HR, hazard ratio; CI, confidence interval; KUGH, Korea University Guro Hospital; KUAH, Korea University Anam Hospital.
